# Case Report: Single-access epidural adhesiolysis and catheter-delivered pulsed radiofrequency for refractory failed back surgery syndrome

**DOI:** 10.3389/fpain.2026.1907035

**Published:** 2026-07-30

**Authors:** Rafael Nascimento

**Affiliations:** 1 Faculty of Medicine, Federal University of Mato Grosso do Sul (UFMS), Campo Grande, MS, Brazil; 2 Pain Medicine Fellowship, Federal University of Mato Grosso do Sul (UFMS), Campo Grande, MS, Brazil; 3 Pain Service, Maria Aparecida Pedrossian University Hospital (HUMAP-UFMS), Campo Grande, MS, Brazil

**Keywords:** dorsal root ganglion, epidural adhesiolysis, epidural catheter, failed back surgery syndrome, pulsed radiofrequency

## Abstract

Failed back surgery syndrome (FBSS) frequently involves mixed mechanical, inflammatory, and neuropathic mechanisms and remains challenging to treat, particularly when systemic anti-inflammatory therapy is limited. We report a 55-year-old woman with chronic refractory left lumbosciatica after endoscopic lumbar discectomy and L3–L4 arthrodesis, with established coronary artery disease and prior myocardial infarction limiting the use of nonsteroidal anti-inflammatory drugs (NSAIDs). Lumbar magnetic resonance imaging demonstrated prior L3–L4 arthrodesis, multilevel degenerative disc disease, and an L4–L5 diffuse disc protrusion causing dural-sac compression, degenerative central canal narrowing, and bilateral foraminal narrowing; lower-limb electroneuromyography did not show active large-fiber radiculopathy. A single-session, single-access caudal procedure was performed using a radiopaque steerable epidural catheter-electrode system, allowing both catheter-directed adhesiolysis and sequential epidural pulsed radiofrequency (PRF) delivery through the catheter tip adjacent to the left S1, L5, L4, and L3 dorsal root ganglion/nerve-root regions. At 5 days, the patient reported approximately 60% improvement in the radiating lower-limb pain. At 8 weeks, she reported approximately 70% improvement in the radicular pain component, while overall pain intensity decreased from NRS 9/10 to 5/10, with residual axial low back pain. A transient sacral neuritis with gluteal paresthesia was the only adverse event and resolved with conservative treatment. This case illustrates a combined interventional strategy for selected patients with refractory FBSS, while the single-case, multicomponent design precludes attribution of benefit to any individual element.

## Introduction

Failed back surgery syndrome (FBSS), also termed post-laminectomy syndrome, denotes persistent or recurrent pain following spinal surgery in the same topographic region. Among its recognized causes—recurrent or retained disc herniation, acquired stenosis, segmental instability, arachnoiditis, and epidural fibrosis—postoperative epidural fibrosis is estimated to account for roughly 20%–36% of cases and correlates with radicular pain and poor surgical outcomes (1).

Percutaneous epidural adhesiolysis (also called percutaneous epidural neuroplasty), introduced by Racz in the late 1980s, addresses this substrate by mechanically disrupting epidural adhesions under fluoroscopic guidance through a steerable catheter, combined with targeted delivery of local anesthetic, corticosteroid, and hyaluronidase, with or without hypertonic saline (2). Randomized and observational evidence supports its short- and intermediate-term effectiveness in refractory post-surgical low back and lower-extremity pain, although the contribution of each individual component remains incompletely defined (1, 3–5).

In parallel, pulsed radiofrequency (PRF) applied adjacent to the dorsal root ganglion (DRG) delivers an electromagnetic field without macroscopic neural injury and has been used as a neuromodulatory option for chronic lumbosacral radicular pain (6–8). Notably, prior lumbar spine surgery has been reported to reduce the therapeutic efficacy of DRG-PRF, which makes a favorable response in the post-surgical setting clinically relevant (9). Isolated reports have described epidural delivery of PRF for persistent radicular pain in FBSS, but combined single-session approaches remain sparsely documented (10, 11). The two techniques target complementary mechanisms—mechanical and chemical lysis of adhesions on one hand, neuromodulation of the sensitized DRG on the other.

A frequent and underappreciated clinical constraint in this population is the need to avoid nonsteroidal anti-inflammatory drugs (NSAIDs), as occurs in patients with coronary artery disease, which narrows the pharmacological options for both the underlying pain and any post-procedural inflammatory response. We describe a single-session combined approach in such a patient, and discuss its rationale, outcome, and limitations.

## Case presentation

A 55-year-old woman presented with chronic, refractory left lumbosciatica of high intensity (numeric rating scale, NRS, 9/10), worse with prolonged sitting and anterior trunk flexion. Her surgical history included endoscopic lumbar discectomy followed by L3–L4 arthrodesis, after which initial relief was transient and the pain recurred and progressed, consistent with post-laminectomy syndrome.

Her medical history was notable for coronary artery disease with a prior myocardial infarction, for which she was receiving single-agent antiplatelet therapy with aspirin 100 mg daily (myocardial infarction in 2022, outside the window for mandatory dual antiplatelet therapy); NSAIDs were avoided because of her established cardiovascular risk. Prior management had failed to provide durable relief and included neuromodulators (pregabalin, duloxetine), a weak opioid (tramadol), cannabidiol, and multiple previous image-guided blocks. There was no relevant family or genetic history, and no relevant psychosocial history was identified.

On examination she was in good general condition, alert and oriented, with thermal and tactile hypoesthesia in the left L5 and S1 territories, a positive left straight-leg-raise and a positive slump test, and preserved motor strength, without sphincteric symptoms. Pre-procedure lumbar magnetic resonance imaging (1.5 T) demonstrated prior L3–L4 arthrodesis with pedicle screws and an intersomatic cage (with hardware artifact), multilevel degenerative disc disease with marginal osteophytes from L1 to S1, and interfacetary arthrosis. A diffuse posterior disc protrusion at L4–L5 caused dural-sac compression with bilateral foraminal narrowing, with left posterolateral protrusions at L2–L3 and L5–S1 and left foraminal narrowing, and mild paravertebral muscle fatty atrophy at L5–S1. The predominantly radicular distribution and this multilevel degenerative substrate informed the targeting of the intervention.

**Figure 1:**
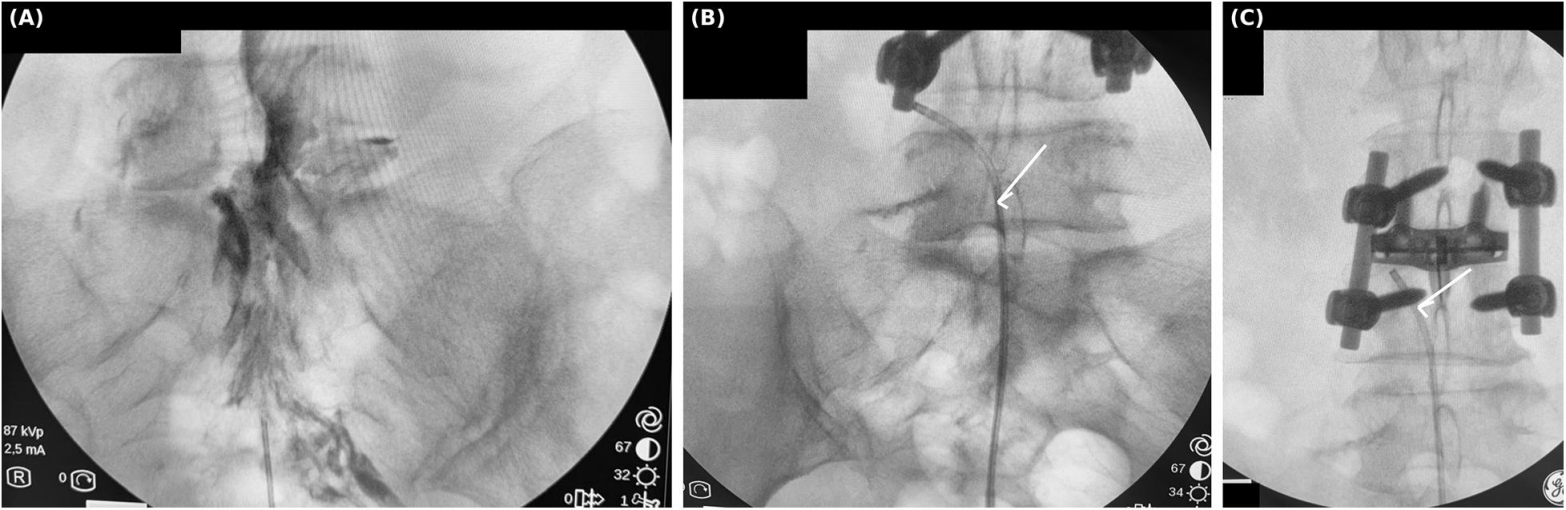
Intraprocedural fluoroscopic images obtained during single-access caudal steerable-catheter epidural adhesiolysis and epidural pulsed radiofrequency. **(A)** Epidurogram with iodinated contrast showing epidural contrast spread in the previously operated lumbar region. **(B)** Radiopaque steerable catheter-electrode (arrow) advanced cranially through the sacral hiatus and navigated toward the operated lumbar segment. **(C)** Anteroposterior view of the instrumented L3–L4 segment showing catheter navigation (arrow) within the operated levels. The same steerable catheter-electrode system was sequentially repositioned to deliver epidural pulsed radiofrequency adjacent to the left S1, L5, L4, and L3 dorsal root ganglion/nerve-root regions. Patient identifiers have been removed.

After cardiology clearance and peri-procedural management of antiplatelet therapy, with aspirin withheld for 7 days before the procedure as advised by cardiology (given the high bleeding-risk classification of epidural adhesiolysis) and resumed after confirmation of adequate haemostasis, a single-session, single-access intervention was performed under sedation in the prone position. Epidural access was obtained through the sacral hiatus using a radiopaque steerable epidural catheter-electrode system (ST.REED PULSE, Seawon Meditech, Bucheon, Republic of Korea), designed to allow both catheter-directed adhesiolysis and epidural pulsed radiofrequency delivery through the catheter tip. Catheter position and epidural spread were confirmed by fluoroscopy and iodinated contrast epidurography (Figure 1). Hyaluronidase-assisted epidural neuroplasty was performed, followed by sequential repositioning of the same steerable catheter-electrode tip toward the left S1, L5, L4, and L3 target regions. At each level, pulsed radiofrequency was delivered epidurally adjacent to the corresponding dorsal root ganglion/nerve-root region using a radiofrequency generator (Cosman G4, Boston Scientific) at 2 Hz with a 20-ms pulse width for 9 min. The output voltage was fixed at 60 V, with 42 °C configured as the predefined temperature ceiling for safety; delivery was therefore voltage-fixed rather than temperature-limited. The output voltage was set as a fixed parameter (60 V), with 42 °C configured concurrently as the temperature ceiling for safety; the voltage was therefore fixed rather than temperature-limited. During application the measured electrode-tip temperature remained below this ceiling, oscillating around 38 °C–39 °C and not reaching 42 °C, consistent with the intended nonablative delivery and the predefined temperature ceiling. The 9-min duration per level was chosen to extend cumulative exposure across the four targeted levels, in line with reported epidural PRF protocols. Motor stimulation at 0.4 V and 2 Hz was used as an additional safety and proximity check before treatment. Segmental injectates and procedural parameters are summarized in Table 1. A total of 80 mg of methylprednisolone acetate was administered during the procedure: 8 mg at each of the four treated left-sided levels (total 32 mg), plus an additional 48 mg diluted in the epidural adhesiolysis infusion with ropivacaine 0.3%. The procedure was completed without intraoperative complications. A sensory stimulation threshold, which is a more specific confirmation of DRG/nerve-root proximity, was not systematically obtained; this is acknowledged below as a limitation.

**Table 1:** Procedure parameters and injectate by level.

Level (left)	Procedure	Hyaluronidase	Epidural PRF (adjacent to DRG)	Injectate
S1	Catheter-directed epidural neuroplasty toward S1	375 IU	42 °C, 60 V, 2 Hz, 20 ms, 9 min	Ropivacaine 3 mg + clonidine 15 µg + methylprednisolone acetate 8 mg (1 mL)
L5	Catheter-directed epidural neuroplasty toward L5/S1	375 IU	42 °C, 60 V, 2 Hz, 20 ms, 9 min	Ropivacaine 3 mg + clonidine 15 µg + methylprednisolone acetate 8 mg (1 mL)
L4	Catheter-directed epidural neuroplasty toward L4/L5	375 IU	42 °C, 60 V, 2 Hz, 20 ms, 9 min	Ropivacaine 3 mg + clonidine 15 µg + methylprednisolone acetate 8 mg (1 mL)
L3	Catheter-directed epidural neuroplasty toward L3/L4	375 IU	42 °C, 60 V, 2 Hz, 20 ms, 9 min	Ropivacaine 3 mg + clonidine 15 µg + methylprednisolone acetate 8 mg (1 mL)

DRG, dorsal root ganglion; PRF, pulsed radiofrequency. Hyaluronidase was prepared by reconstituting a 3,000 IU vial in 2 mL; 1 mL (1,500 IU) was then rediluted in 10 mL and distributed equally across the four treated levels (375 IU per level; total 1,500 IU). The methylprednisolone acetate dose shown in the table refers to the segmental injectate administered at each treated level. An additional 48 mg of methylprednisolone acetate was administered in the epidural adhesiolysis infusion (with ropivacaine 0.3%), resulting in a total intra-procedural dose of 80 mg.

## Outcome and follow-up

Five days after the procedure the patient reported approximately 60% reduction of the radiating lower-limb pain. She additionally developed acute sacral pain with gluteal paresthesia, worse on sitting, without motor deficit, sphincter dysfunction, or symptoms suggestive of cauda equina syndrome. This was interpreted as a transient post-manipulation sacral neuritis, likely related to adhesiolysis and a local inflammatory response. Because systemic NSAIDs were avoided owing to her cardiovascular risk, management was conservative, with oral analgesia and physical measures; the picture was self-limited.

**Figure 2:**
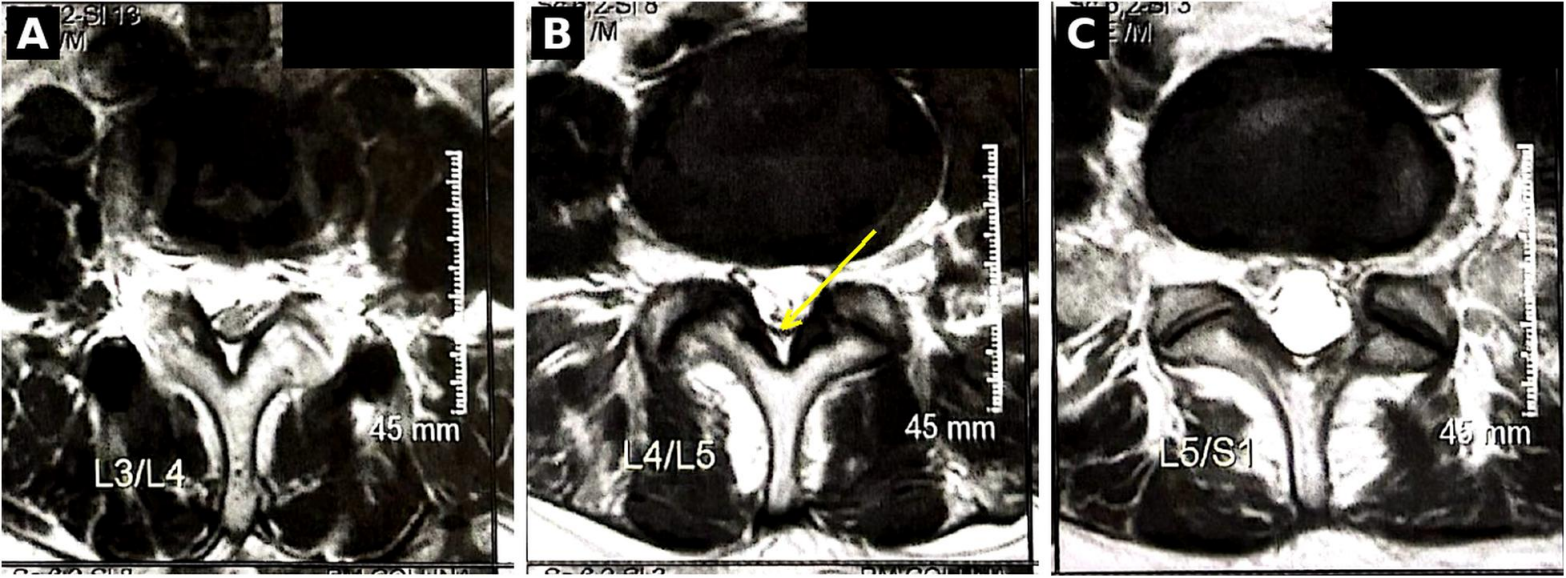
Axial T2-weighted lumbar MRI obtained after the procedure (21 April 2026) at the operated and adjacent lower lumbar levels. **(A)** L3–L4; **(B)** L4–L5, showing degenerative central canal narrowing (arrow) with facet hypertrophy and ligamentum flavum thickening (cross-sectional area approximately 119 mm^2^, reported as borderline and essentially unchanged compared with the prior 2024 examination); **(C)** L5–S1. The multilevel degenerative substrate is consistent with the persistent axial component. Patient identifiers have been removed.

At 8-week follow-up, the patient reported approximately 70% improvement in the radiating lower-limb pain component. Overall pain intensity decreased from NRS 9/10 before the procedure to NRS 5/10, with persistent residual axial low back pain, particularly while sitting (Figure 2). No new neurological deficit was observed. A standardized functional measure (e.g., Oswestry Disability Index) was not formally documented, which is acknowledged below as a limitation. Reporting the two components separately, the radicular (leg) component improved by approximately 60% at 5 days and 70% at 8 weeks, whereas the axial (low back) component remained at NRS 5/10; standardized patient-reported instruments (e.g., PGIC) and a formal record of analgesic consumption were not systematically collected, and rescue-analgesic use decreased qualitatively over follow-up. The overall timeline of the episode of care is summarized in Table 2.

**Table 2:** Timeline of the episode of care.

Time point	Event
Prior years	Endoscopic lumbar discectomy
Subsequently	L3–L4 arthrodesis (with transient relief, then recurrence)
Pre-procedure	Refractory left lumbosciatica, NRS 9/10; failure of pharmacological and interventional management
Procedure day	Caudal steerable-catheter epidural adhesiolysis + left S1/L5/L4/L3 DRG-PRF, under sedation
Day 5	∼60% improvement in radicular pain; transient sacral neuritis with gluteal paresthesia
Week 8	∼70% improvement in radicular component; overall pain NRS 5/10; residual axial low back pain

NRS, numeric rating scale; DRG-PRF, epidural pulsed radiofrequency delivered adjacent to the dorsal root ganglion/nerve-root region.

## Patient perspective

The patient reported that the reduction in radiating leg pain was clinically meaningful and improved her tolerance to daily activities. She also noted that axial low back pain persisted, especially while sitting, and that the transient sacral/gluteal discomfort after the procedure was self-limited.

## Discussion

This case illustrates a single-session combination of percutaneous epidural adhesiolysis and multilevel epidural PRF delivered adjacent to the DRG/nerve-root region (DRG-PRF) in a patient with refractory FBSS whose management was further constrained by the avoidance of NSAIDs because of cardiovascular risk. Two features make the case noteworthy. First, the response was obtained in a post-surgical patient—a subgroup in which DRG-PRF has been reported to be less effective than in non-operated patients (9)—yet a clinically meaningful reduction in radicular pain was achieved over the 8-week follow-up. Second, the avoidance of NSAIDs, common in patients with cardiovascular disease, removed a standard tool for managing both the baseline pain and the expected post-procedural inflammatory response, increasing the value of a durable interventional result.

The biological rationale for the combination is complementary. Adhesiolysis aims to mechanically and chemically release epidural scar tissue thought to tether and sensitize nerve roots, restoring the local environment and improving the distribution of injected agents; hyaluronidase facilitates this by degrading the extracellular matrix of adhesions. DRG-PRF, in turn, is proposed to modulate the sensitized ganglion through non-thermal electromagnetic effects, including attenuation of microglial activation and pro-inflammatory cytokine signaling, without structural neural damage (7, 9). Targeting both the mechanical substrate and the neuromodulatory target in a single session is a pragmatic strategy in highly refractory disease.

The findings should be interpreted with considerable caution. As a single case (*n* = 1), no causal inference is possible. The intervention was deliberately multi-component—mechanical adhesiolysis, hyaluronidase, corticosteroid, and DRG-PRF at four levels—so the observed benefit cannot be attributed to any individual element; the epidural corticosteroid alone (a total of 80 mg methylprednisolone acetate, a substantial dose in a previously NSAID-constrained patient) could plausibly account for much or all of the early radicular improvement; clonidine, included in the segmental injectate, has its own analgesic and anti-hyperalgesic action and adds a further uncontrolled variable; and natural history, regression to the mean, or a placebo effect may each also have contributed. The follow-up was short (8 weeks), and a validated functional outcome (Oswestry Disability Index), a sensory stimulation threshold before PRF, and standardized outcome instruments (PGIC, quantified analgesic use) were not formally recorded, limiting assessment beyond pain intensity. Finally, the transient sacral neuritis observed underscores that the combined approach is not without self-limited adverse effects, the management of which was itself constrained by the avoidance of NSAIDs.

Within these limitations, the case supports the consideration of a combined adhesiolysis plus DRG-PRF strategy as one option in selected patients with refractory FBSS, particularly when anti-inflammatory pharmacotherapy is limited. Prospective, controlled studies designed to isolate the contribution of each component, with standardized functional endpoints and longer follow-up, are needed before any efficacy claim can be made.

## Data Availability

The original contributions presented in the study are included in the article/Supplementary Material, further inquiries can be directed to the corresponding author.
